# Using machine learning to automate the collection, transcription, and analysis of verbal-report data

**DOI:** 10.3758/s13428-025-02800-5

**Published:** 2025-09-12

**Authors:** Tehilla Ostrovsky, Paul Ungermann, Chris Donkin

**Affiliations:** 1 School of Psychology, Ludwig Maximilian University, Munich, Germany; 2 Technical University of Munich, Munich, Germany

**Keywords:** Natural language processing models, Self-report, Verbal description, Decision-making, Cognitive model validity

## Abstract

What people think and say during experiments is important for our understanding of the human mind. However, the collection and analysis of verbal-report data in experiments are relatively costly and are thus grossly underutilized. Here, we aim to reduce such costs by providing software that collects, transcribes, and analyzes verbal-report data. Verbal data are collected using jsPsych (De Leeuw, *Behavior Research Methods*, *47*, 1–12, 2015), making it suitable for both online and lab-based experiments. The transcription and analyses rely on classical machine-learning methods as well as deep learning approaches (e.g., large language models), making them substantially more efficient than current methods using human coders. We demonstrate how to use the software we provide in a case study via a simple memory experiment. This collection of software was designed to be modular, allowing for the update and replacement of various components with superior models, as well as the easy addition of new methods. It is our sincere hope that this approach popularizes the collection of verbal-report data in psychology experiments.

**Supplementary Information:**

The online version contains supplementary material available at 10.3758/s13428-025-02800-5.

## Introduction

Among experimental psychologists, self-report has a relatively bad rap. Compared to measures like choice proportions, accuracy, and reaction times, explicit verbal reports are often seen as subjective and noisy, both in terms of what participants produce and also in how they are analyzed. Two key properties of verbal reports give rise to this reputation. The first is that people do not have perfect insight into the reasons they do things, which was a major impetus for the cognitive revolution. Second, people can express themselves in a variety of ways, which has typically necessitated the need for verbal responses to be analyzed by other human coders subjectively and inefficiently.

While there is no doubting that introspection can be problematic, it is becoming increasingly clear that we have overcorrected and that largely ignoring what people say they are doing is holding us back. For example, Frey et al. (2017) compared behavioral measures from controlled experimental psychology tasks with self-reported questionnaires on risk-taking. They showed that self-report tasks frequently outperform behavioral tasks in assessing and predicting risk preferences. Responses to questions, such as ’Are you generally a risk-taking person or do you try to avoid risks?’ and ’How likely would you be to go white-water rafting at high water in the spring?’, not only better reveal stated preferences than choice proportions in experimental tasks, but they also correlate with specific real-world risky activities. Such a result likely reflects a general trend, given the slew of recent reports of poor test-retest reliability in measures taken from experimental tasks  (Enkavi et al., 2019; Hedge et al., 2018; Rouder & Haaf, 2019).

Many cognitive theories also clearly imply what people *should* be able to (and not able to) say while performing a task. Ostrovsky et al. (2024) argue that explicit verbal reports can be used to test such theoretical claims. Such a development is welcome, since implications about what should be in the minds of participants are rarely tested, and so are a neglected source of flexibility in our cognitive theories (Szollosi & Donkin, 2019). Indeed, we suspect that for the vast majority of phenomena in psychology, it is so obvious that we need to understand what people can and cannot say while performing a task, that the major reason that most researchers ignore verbal reports is pragmatic. That is, because collecting and analyzing verbal data is simply much too challenging.

There are many ways of eliciting people’s thoughts or reasoning during a task. Free-text or verbal reports can refer to the articulation of a person’s thoughts, feelings, attitudes, or cognitive processes in response to specific stimuli or tasks. The primary aim of verbal reports is to gain a more granulated insight into the cognitive processes underlying behavior, decision-making, problem-solving, and other psychological phenomena. These reports may be spoken aloud or provided in written form. In short, collecting verbal report data requires participants to verbalize their thoughts, either in real-time while engaged in a specific task (e.g., using a ’think aloud’ method), or retrospectively after the task is complete.

Many authors have written extensively about best practice and what should be considered when collecting verbal data (e.g., Ericsson & Simon, 1980; Fox et al., 2011; Ranyard & Svenson, 2011; Svenson, 1979, 1989). Many such concerns are around what Ericsson and Simon (1980) called the suitability of the task: determining whether and how to elicit verbal reports during the task. We leave it to the Discussion section to address this problem, since the details will inevitably be specific to the research question at hand. Rather, our goal in this manuscript is to deal with the second major issue that Ericsson and Simon address, the challenge of scalability.

Verbal report data can be large, messy, subjective, sometimes irrelevant, overwhelming, and thus, costly. The usual method for analyzing verbal report data is via a human coder/rater. A researcher is typically trained to condense the text, for example, by creating shorter summaries or by classifying responses into certain types. This approach is expensive, usually in terms of both time and money, especially since multiple raters will often be used to check whether the coding is reliable.

The cost of verbal-report data is, we suspect, mostly responsible for its lack of use. For example, participant numbers in experiments must be kept relatively small, since the cost grows larger with increased sample sizes. As such, concerns about the variability in verbal-report data are reasonable – simply collecting more data to increase the signal relative to the noise is rarely an attractive option. Furthermore, the cost makes it daunting for an experimental psychologist to incorporate verbal report data into their own experimental paradigms, since it is not always clear what participants might say, and coders must be given specific instructions when analyzing text (e.g., what types of responses to anticipate classifying).

### Using large language models to overcome the challenges with verbal reports

To address these challenges with the scalability of verbal reports, we follow the proposal in Ostrovsky et al. (2024), to use large language models (LLMs). At the time of writing, LLMs are typically of the transformer architecture (Vaswani et al., 2017), such as GPT (generative pre-trained transformer) models (Brown et al., 2020; Achiam et al., 2023), BERT (bidirectional encoder representations from transformers) models (Devlin et al., 2019) and BART (bidirectional and auto-regressive transformers) models (Lewis et al., 2019). Such models bring several advantages when analyzing verbal data. Their architecture and training enable them to represent words and sentences in a way that incorporates semantics and context, allowing them to handle the nuanced expressions found in ’think-aloud’ data. As such, LLMs are able to perform relatively well at many of the kinds of tasks that are usually given to human coders, such as summarizing and classifying text.

The way by which LLMs analyze verbal data is primarily achieved through their embeddings – high-dimensional num-erical representations for words or sentences that encode their semantic meaning in a given context. These embeddings are similar to the data created by other tracing methods used in psychological research, such as eye fixation patterns or EEG signals. In the same way, we can analyze these embeddings to reveal structure and systematicity in the numerical representati-ons, and then look at what this implies for the text that produced them. That is, the LLMs translate between text and num-bers, allowing us to use the methods developed for finding patterns in numerical data to find patterns in text. This approach seems to work. Indeed, by now, anyone who has used an LLM knows that it is capable of performing many of the tasks that we would give to a human rater of verbal report data.

**Fig. 1:**
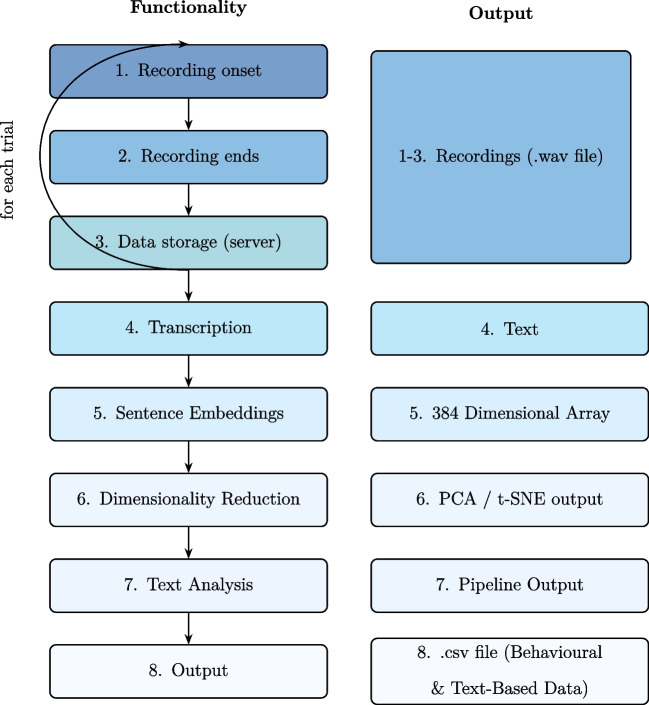
Flowchart describing the timeline of the functionalities and the outputs of the framework. Boxes 1–3 represent the recording of each trial. Box 4 shows the transcription of the recorded trials into text. Box 5 represents the conversion of the text data into embeddings. Box 6 illustrates the step of the dimensionality reduction performed on the embeddings from Box 5. Box 7 represents the pipelines applied to the transcribed text from Box 4’s output, and Box 8 shows the final output after all processes from Boxes 1–7 have been applied

Since LLMs seem to be able to approximate human raters, their most attractive property is that they can process large amounts of text data swiftly and relatively cheaply. It is hard to overstate the potential that such a benefit over traditional qualitative methods could offer. The relatively small cost of using LLMs to analyze verbal data should solve many of the aforementioned barriers to including verbal reports in experimental psychology. For example, researchers need not anticipate what kinds of things participants will say during an experiment, but can rather run and rerun various analyses to discover the kinds of things people said. The scalability of LLMs analyses also means that the amount of verbal report data that researchers can collect and analyze can also now grow to sizes like we are used to with other dependent measures. Not only does this help increase the reliability of the data we collect, but it also gives us another way to deal with the noisiness of verbal data.

The aim of this article is to provide software that makes it easy for researchers to collect spoken verbal reports, to automatically translate those reports into text, to transform that text into numerical embeddings using an LLM, and to then apply a suite of analyses of the resultant quantitative data. Given the tremendous pace with which this field is moving, the software is designed to be easily changed to incorporate newer methods and models. As such, we refer to it throughout as a ’framework’ rather than a particular piece of software.

Lastly, our framework builds on preexisting libraries and pre-trained models, it offers an automated pipeline for continuous audio recording and precise segmentation of verbal reports into trials, its integration with the widely used jsPsych platform allows for both online and offline experimentation across a range of research domains. The framework’s ready-to-use implementation within jsPsych, along with provided example scripts for model fine-tuning and direct interaction with large language models, hopefully makes it easier for psychology researchers to incorporate these relatively novel techniques. Most importantly, by significantly lowering the barrier to using verbal reports in experimental designs, this out-of-the-box framework has the potential to open up their use to a wider audience, ultimately driving progress in our study of behavior and cognition.

## Framework

The core role of the framework we propose here is to record participants’ spoken reports during a study, automatically translate them into text, and analyze that text using various machine-learning-based analyses. The framework is modular and subject to updates as desired. For a schematic description of the framework, see Fig. 1.

To give an overview, the recording aspect of the framework is implemented in jsPsych (De Leeuw, 2015), a popular JavaScript-based framework to conduct studies in a web browser, which should allow it to be easily integrated into the kinds of studies already run using jsPsych. While the recording occurs continuously throughout the experiment, what is said is segmented into trials as per usual within jsPsych (Boxes 1–3, Fig. 1). Both the recordings and the corresponding behavioral data (the default jsPsych output) are transferred to a server for storage.

Once the recordings are saved, a deep-learning-based transcription model is used to convert the audio recordings into written text for further analysis (Box 4, Fig. 1). For this, we provide a platform-independent open-source solution that is ready to use, along with an in-depth tutorial on how to integrate it into a new study^1^. Finally, we provide a set of possible analyses that can be adapted to be run on written text. Most of these analyses rely on embeddings created by an open-source LLM (Box 5, Fig. 1). A series of subsequent analyses are provided (Boxes 6–8, Fig. 1), though this is where we expect the exact contents to be readily modified and expanded by the users.

Our choice of models was guided by three main considerations: data privacy, reproducibility, and computational efficiency. Running models locally helps ensure that sensitive participant data remains secure within the research environment. An additional advantage of using open-source models (i.e., models with fixed publicly available weights) is that they produce deterministic outputs, which is essential for reproducibility. Unlike proprietary models, which are frequently updated without public disclosure, open-source models offer greater transparency. Open-source models, on the other hand, typically remain stable over time, and any modifications are openly documented and accessible to users. Lastly, the relatively small size of the models we used reduces computational demands and overall costs, making the framework more accessible and scalable.

We now provide a detailed description of each of these functionalities in turn. Then, in the “Case study” section, we demonstrate and evaluate the performance of each of these functionalities.

### Recording

The initial step within the framework involves the recording of participants. This functionality is activated at the beginning of each trial. As illustrated in the code snippet 1, the jsPsych function onTrialStartRecording is invoked to initiate the recording process. The recording process concludes at the end of each trial with the function onTrialFinishRecording. These functions activate the recordings locally on the person’s computer (see top 3 left rectangles Fig. 1). At the end of each trial, the recording is sent back to the server and is saved as a .wav file (see the top right rectangle in Fig. 1).

Upon completion of the experiment, to ensure participants have the option to defer their participation, they are asked whether they still consent to the transmission and analysis of their data. If they decline, their data is immediately deleted from the server. Similarly, if they leave the experiment early, their data is deleted.
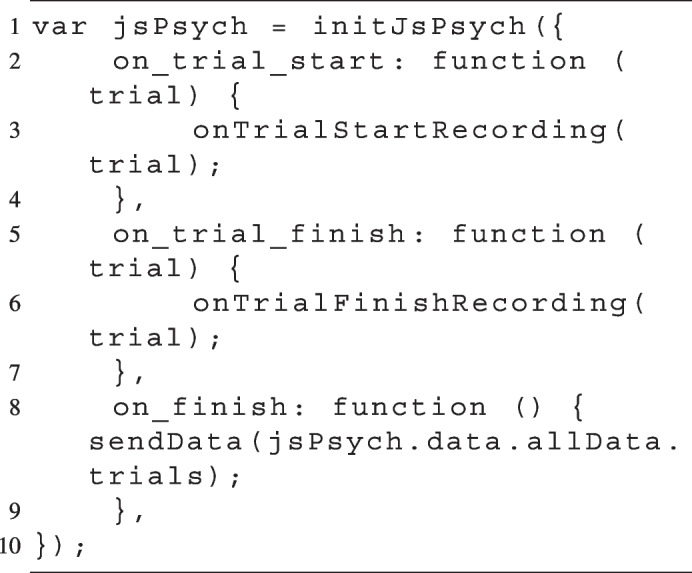


#### Storage requirements

To provide readers with a sense of the storage requirements involved, one minute of spoken verbal reports typically results in approximately 10 MB of audio data. For instance, an experiment involving 90 participants with 100 trials each – assuming an average recording length of 30 s per trial – would generate an estimated 45 GB of data. It is therefore important that researchers evaluate whether their available storage infrastructure is sufficient to accommodate the anticipated data volume.

For users working with large volumes of data that may challenge local computing capabilities, we recommend two secure and scalable alternatives. First, users can utilize commercial cloud platforms such as Amazon’s AWS or Google Cloud. These services provide secure, paid environments with multiple layers of data protection, including physical security at data centers, end-to-end encryption, and comprehensive compliance with international standards and certifications. Alternatively, users may opt for storage solutions provided by their universities or research institutions. These university-managed private cloud services offer a secure infrastructure tailored to academic research needs and often comply with internal data protection policies and ethical standards.

**Table 1:** Comparison of different transcription models

Model	Accuracy (WER)	Cost	Speed	Open-source	Local?
Google Speech-to-Text	18.9%	0.016$/min	1149.1s	no	no
Assembly AI	14.7%	0.002$/min	143.2s	no	no
Deepgram’s Nova-2	13.2%	0.0043$/min	29.8s	no	no
Whisper	12.8%	(0.006$/min)	229.6s	yes	both

Note. Accuracies taken from Dilmegani and Alper (2025). The speed is the median inference time for one hour of audio using the API, taken from Fox and Francisco (2025). The speed of Whisper may vary depending on the hardware when running locally

### Transcription

Once the full recordings are stored on the server, experimenters can proceed to send these recordings for transcription, transforming the audio recordings into text. The output of this step is the transcribed text for each trial. Examples of such transcribed recordings are shown in Table 3 under the column *transcribed text*. In the current version of the framework, this step is done using OpenAI’s Whisper deep-learning model (Radford et al., 2022a), chosen for its (at the time) state-of-the-art performance in speech-to-text tasks and its open-source availability (see box 4 in Fig. 1). At the time of writing the *large* version of the model had 1.54 B parameters (more information about this model can be found at huggingface.co/openai/whisper-large). As these models are constantly updated, the specific model version is a parameter in our framework and can be adjusted in the script under  within the configuration file (which can be found at github.com/tehillamo/AutoV-LLM/blob/main/data_pipeline/config.json).

Table 1 provides a comparison of several alternative transcription models available at the time of writing. While these specific approaches are likely already outdated by the time of publication, key features such as the model’s accuracy, its associated cost, its speed, and open-source availability are likely to remain generally relevant. These criteria can continue to serve as useful guidelines for researchers when evaluating and selecting transcription models for their own work. We, therefore, encourage readers to explore these options when deciding on the best fit for their project.

#### Processing time

It is worth taking a moment to give an indication of how long the transcription process currently takes to run, noting that this is the most computationally intense component of the current version of our analysis pipeline. Using the previous numbers of 90 participants per 100 trials and 30 s for each verbal report, we get 9000 lines of data and 75 h of recording. Using typical cloud-based computing hardware, Whisper takes about 5 h to transcribe this into text. So, transcribing such text would currently cost about USD$5 on Google Cloud, as an example. Running the same transcription on a typical consumer-grade personal laptop would take about 20–25 h.

### Embeddings

The next step in this framework involves generating text embeddings for each trial, based on the transcribed text (see box 5 in Fig. 1). Embeddings are numerical representations of text, produced by transforming words or phrases into vectors of real numbers. These high-dimensional embedding vectors encode the meaning of a given piece of text in its given context. Sentence embeddings are specifically designed to capture semantic similarity, ensuring that semantically similar sentences are positioned closer together in the vector space. As such, the embeddings facilitate a range of analyses and visualization techniques that can help a researcher to summarize and understand what was spoken.

We compute these embeddings using the Sentence Transformer ’all-MiniLM-L6’ (Reimers & Gurevych, 2019), which is an open-source model, is relatively small, is fast to use, while still offering good performance relative to larger models (more information about this model can be found at  https://huggingface.co/sentence-transformers/all-MiniLM-L6-v1). This version of the model has 22.7 million parameters and outputs a 384 dimensional embedding vector. As these models are constantly updated, this model can be changed in the script under  within the  file (which can be found at  github.com/tehillamo/AutoV-LLM/blob/main/data_pipeline/embeddings.py).

It is noteworthy that this length is specific to our model; other models produce embeddings of varying dimensions. For example, OpenAI’s embedding "text-embedding-ada-002" produces 1536 dimensional vectors, while RoBERTa(Liu et al., 2019) generates 768 dimensional embeddings. These differences arise due to variations in design choices (the choice of how many dimensions there should be) and application (e.g., an upstream task).

**Table 2:** Comparison of different embedding models.

Model	Score$\uparrow$	#Parameters	Embedding size
multilingual-e5-large-instruct (Wang et al., 2024)	63.2	560M	1024
GritLM-7B (Muennighoff et al., 2025)	60.9	7B	4096
e5-mistral-7b-instruct (Wang et al., 2023)	60.3	7B	4096
multilingual-e5-large (Wang et al., 2024)	58.6	560M	1024
multilingual-e5-base (Wang et al., 2024)	57.0	278M	768
multilingual-mpnet-base (Reimers & Gurevych, 2020)	52.0	278M	768
multilingual-e5-small (Wang et al., 2024)	55.5	118M	384
LaBSE (Reimers & Gurevych, 2020)	52.1	471M	768
multilingual-MiniLM-L12 (Reimers & Gurevych, 2020)	48.8	118M	384
all-mpnet-base (Reimers & Gurevych, 2020)	42.5	109M	768
all-MiniLM-L12 (Reimers & Gurevych, 2020)	42.2	33.4M	384
all-MiniLM-L6 (Reimers & Gurevych, 2020)	41.4	22.7M	384

Data taken from MMTEB (i.e., Massively multilingual text embedding benchmark) Enevoldsen et al. (2025)

Table 2 presents a comparison of several alternative *embedding* models evaluated using the Massively Multilingual Text Embedding Benchmark (MMTEB).^2^ This benchmark assesses open-source embedding models across a variety of tasks, datasets, and languages, using a custom scoring system that integrates multiple evaluation metrics. For detailed methodology and results, please refer to the original MMTEB paper  Enevoldsen et al. (2025). We again note that while these alternative *embedding* models are available at the time of writing, their availability and the relative quality of their performance will change in the future. However, the key features highlighted in this table – such as language support, model size, open-source availability, and general benchmark performance–are likely to remain central considerations. We encourage readers to stay informed about recent developments and use these features as a guide when selecting an appropriate model for their research.

Lastly, the trade-offs between model size, computational requirements, and performance guided our choice of the all-MiniLM-L6 model. This model has been widely adopted in the research community for its strong reputation in offering a well-balanced compromise between efficiency and accuracy. Moreover, it is important to emphasize that the effectiveness of an embedding model is highly task-dependent, and the right choice may vary depending on the specific goals and constraints of the research.

### Dimensionality reduction

The first analysis of embeddings we provide (Box 6 in Fig. 1) attempts to reduce the high-dimensionality of the embeddings to determine if the text varies on any systematic, meaningful psychological concepts (such as “confidence”, “context”, or “riskiness”). Such a reduction can be useful because the embeddings are of such high-dimensionality, making the problem similar to those in other psychological research fields, where things like principal component analysis (PCA) or factor analysis are used to simplify data. The idea underlying dimensionality reduction methods is that the text embeddings may vary in such a way that, for a particular experiment, there is a small number of frequently occurring features of the text, such as statements about confidence or risk, that account for most of the variability in what was said by participants.

We have implemented two techniques for dimensionality reduction: PCA and the t-distributed stochastic neighbor embedding (t-SNE) (Van der Maaten & Hinton, 2008). We offer PCA as it is a historically popular method of dimensionality reduction in a number of fields of psychology. We also offer an alternative, t-SNE, which is similar but reduces the dimensionality of the data while preserving the data’s neighborhood structure. This technique is, at the time of writing, popular for use with text embeddings.

Users can modify the method for dimensionality reduction to either "PCA", "t-SNE", or "both" in the config.json script under:  . Both methods require the user to specify the number of dimensions to which the raw embeddings should be reduced, and while the default setting is 2, this can be configured by changing the line:  in the same configuration file. Finally, the analyses provided by default apply the reduction to all participants and trials simultaneously, and not on a participant-by-participant basis, but this can also be changed in the config.json file by modifying the  option from false (the default) to true.

The output of this functionality is, for both methods of reduction, the position of each trials’ text in the new, reduced space. For instance, if the experimenter selects the default option in our framework, which compresses the raw, high-dimensional embeddings array to two dimensions, the output for each trial will be a two-dimensional vector representing a compressed numeric representation of the sentence.

### Text analysis

Box 7 of Fig. 1 is a catch-all term for the variety of analyses one might use to extract meaningful insights from transcribed text. By default, we have provided a text classification algorithm that allows users to categorize each verbal report into predefined labels, as well as a keyword extraction algorithm (for more details see  Grootendorst (2020a)), and an auto-summarization method. These tools are just some of the techniques that researchers can use to distill substantial information from text. Below, we provide details on each of these default pipelines.

It should be noted that this aspect of the framework is intentionally flexible because the appropriateness of the different pipelines is context-dependent and must be carefully chosen by the researcher. For example, summaries may not be practical for brief transcriptions, and a lot of work may have to go into creating adequate labels for labeling techniques. As a default, these methodologies are included in the output; however, the reader should not take this as a sign of endorsement for their use. Rather, we expect users to select and modify these tools as needed. Indeed, it bears repeating that due to the rapidly evolving machine-learning landscape, the entire framework is modular so as to facilitate easy substitutions, such as switching between the choice of particular models (both for transcription and for the embeddings) and between text analyses methods (for more details, see our documentation at https://github.com/tehillamo/AutoV-LLM/).

#### Text labeling

One way to understand verbal report data is to find an appropriate label for each utterance. We use a zero-shot classification algorithm to identify the most likely text label for a given input. The text labeling employs a “zero shot” learning pipeline to assign labels to participant-recorded sentences  (Yin et al., 2019; Sanh et al., 2022). This method has the benefit of not requiring training with specific labels; instead, it uses the set of labels provided by the user to classify the text according to how it was trained, generically speaking. A score is given to each label, indicating how well it matches the input text (in the space of high-dimensional embeddings). The label with the highest score is considered the most appropriate label.

In this pipeline, users can modify the set of labels to their needs by adjusting the text_classes variable (text_classes = ["label_1", "label_2"]) in the configuration file and change the classifier with pipe = pipeline(model="facebook/bart-large-mnli") in the keyword_similarity_zero_shot function, found in the text_classification.py script within this GitHub repository: https://github.com/tehillamo/AutoV-LLM/blob/main/data_pipeline/text_classification.py. By default, the output is the most appropriate label, but the code can be altered to also yield the score for all of the candidate labels. The line of code responsible for selecting and saving the label with the highest similarity in the output file is located in the  script. This line of code is:



Users who wish to save the entire list of labels, along with their associated scores, can remove or comment out this line. If users only want labels that surpass some level of appropriateness (or confidence, per the nomenclature in the field), then they can set a threshold in the configuration file  under  . Smaller threshold settings (e.g., .1) will lead to more labels being considered appropriate, while higher thresholds (e.g., .3 or above) return fewer labels, which can improve accuracy but may lead to some appropriate labels being missed.

#### Keyword extraction

Keyword extraction is the automated process of identifying the most relevant words and phrases from an input text. We use the *BERT* model, a bi-directional transformer that converts phrases and documents into vectors that capture their meaning  (Grootendorst, 2020b). KeyBERT  (Grootendorst, 2020a) is a simple and effective keyword extraction technique that utilizes BERT embeddings to generate keywords and key phrases most closely related to the document (for details about this model, see  https://github.com/MaartenGr/KeyBERT). As with other pipeline scripts, the model used can be modified in the keywords.py script by adjusting the variable  found in the repository at https://github.com/tehillamo/AutoV-LLM/blob/main/data_pipeline/keywords.py. The output of this algorithm is currently set to a default of 5 keywords, as defined by the argument  in the function configuration file. Researchers can adjust this value as needed.

#### Auto-summarization

For text summarization, we use the BART pipeline, which is a sequence-to-sequence model, meaning that it is a type of deep learning model specifically designed to handle tasks where the input and output are both sequences of data. At the time of writing, the model has 400 million parameters (for more details, refer to https://github.com/facebookresearch/fairseq/tree/main/examples/bart), which it uses to shorten a given text input down to a specified length, while preserving the most important features of the input text.

As with any other text analysis pipeline, this model can be rep-laced with another in the  script by modifying the line  , available in our repository at https://github.com/tehillamo/AutoV-LLM/blob/main/data_pipeline/summarization.py. The output from the summarization analysis consists of summaries for each verbal report, provided they are sufficiently lengthy to warrant summarization. The current settings limit the summary length to between 30 and 130 words. These parameters can be adjusted by modifying the  and  arguments in the config file.

#### Fine-tuning for zero-shot classifier

Fine-tuning models for zero-shot classification provides both more accurate and better adapted results to the specific language of the task. To support users in implementing this approach, we provide a second script for fine-tuning a pre-trained *BART-NLI* model (facebook/bart-large-mnli) on domain-specific data (see https://github.com/tehillamo/AutoV-LLM/blob/main/data_pipeline/example_scripts/zero_shot_finetuning.py). In our case, the dataset includes verbal reports about memory and confidence, allowing the model to learn how people typically express certainty. As a result, the model becomes better at classifying new, unseen reports based solely on language, even when the phrasing or labels differ. For example, training on sentences like “I remember it clearly” (very sure),“I could be wrong” (not sure), and “I think it was ...” (somewhat sure) enables the model to recognize and accurately classify similar expressions, such as “I’m fairly certain I saw the word A, but I wouldn’t swear to it..” Fine-tuning in this way allows the model to perform more reliably and flexibly in zero-shot classification settings. Practically, the script takes a user-provided dataset in CSV format, tokenizes the text using the *bart-large-mnli* tokenizer, and turns the labels into numeric values for training. It then fine-tunes the model using the *Hugging Face Trainer API*. Once training is finished, the model is saved for future use in text classification tasks.

### Optional extensions: Direct use of large language models

Once the transcription work is complete, an alternative approach to text analysis is to make direct use of large language models (LLMs) and, where appropriate, fine-tune them for specific research contexts. For example, one might provide ChatGPT with the verbal reports from a participant and ask it to provide a brief summary of the spoken text. Through careful engineering of the specific prompt provided to the LLM, these models can provide all sorts of valuable outputs (see Bunt et al. (2025) for discussion and advice on the development of such prompts).

The main upside of direct use of an LLM is that it may offer significant advantages in terms of semantic depth and contextual nuance Bunt et al. (2025), however, this method can be computationally demanding and introduces some new concerns over data privacy. Our focus in this paper is on open-source tools to promote accessibility, reproducibility, and data privacy, we acknowledge the increasing value of integrating LLMs more dynamically into text analysis workflows. For researchers equipped with the necessary resources, this approach opens up a wide swath of possibilities.

To support those interested in such analyses, we have included example scripts in our GitHub repository that demonstrate how to generate embeddings directly using OpenAI’s API (see https://github.com/tehillamo/AutoV-LLM/blob/main/data_pipeline/openai_script.py), as well as how to fine-tune a model (in our example, the sentence-transformer *SentenceBERT*) on domain-specific data (see https://github.com/tehillamo/AutoV-LLM/tree/main/data_pipeline/example_scripts). These ex-amples are intended to help researchers adapt and expand the framework to suit their own datasets and research questions. While we encourage the community to explore this powerful extension of the workflow, in the remainder of this paper, we will continue to focus on the core open-source models, which are designed to be fully functional and accessible without requiring advanced infrastructure or proprietary tools.

### Output

The final step in the framework is to merge all of the outputs computed in each of the above sections, including the transcribed text, embeddings, and the results of the included analyses of the text, with the behavioral data obtained from the actual study. This process results in a .csv file with each feature, such as the transcribed text, presented in its own separate column (see Box 8 in Fig. 1). For a truncated example, see Table 3. The script that merges all data together can be found and altered in https://github.com/tehillamo/AutoV-LLM/blob/main/data_pipeline/merge_behavioral_data.py.

**Table 3:** Sample data from our machine learning pipeline

Trial number	Transcribed text	rt	Text embedding
100	West and geese I don’t	10532	[5.8e-02, -2.5e-02, -1.0e-02, 2.0-02,<...>]
104	Pretty sure it’s spout.	10113	[-1.8e-02, -4.3e-02, -3.8-02, 2.2e-02<...>]
520	I think Canada was there	3092	[9.6e-02, 9.6e-02, -3.2e-02, -9.2e-03<...>]
516	China or geese	18038	[1.5e-02, 5.2e-02, -1.7e-02, 6.5e-02<...>]
496	Float. Pretty sure. Float.	5669	[1.9e-02, 1.7e-02, -8.5e-02, 3.8e-02<...>]
292	I don’t remember what I said.	14267	[-7.6e-02, 3.0e-03 4.4e-02, 1.2e-01<...>]
484	Sweden I do have	2836	[6.7e-02, 4.3e-02, -2.5e-02, -4.0e-02<...>]
448	I do remember seeing Taiwan.	5540	[9.6e-02, 4.1e-02, -1.2e-02, 4.5e-02<...>]

Note: The *trial number* and *rt* columns show the trial number and reaction time recorded by default in the jsPsych framework. The *transcribed text* column contains the transcription of verbal reports, and the *text embedding* column represents the embeddings of those transcriptions for each trial, both generated by our framework. The transcribed text is truncated due to space limitations, and we selected only a few rows for better visualization (refer to the trial number column)

**Fig. 2:**
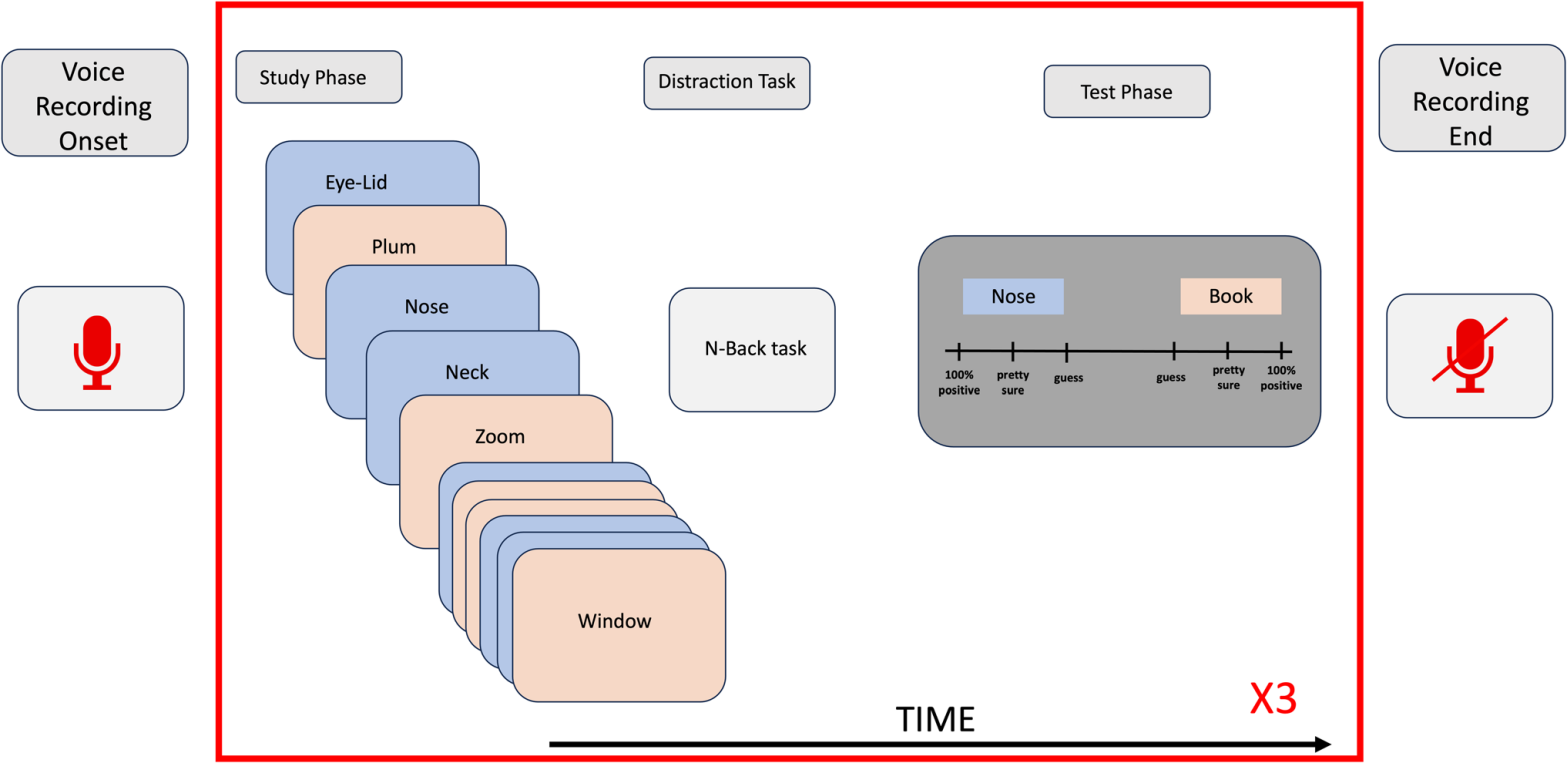
An example of a trial’s timeline. Note. The experiment began with asking for permission from the participant to activate their microphone. After obtaining consent, the instructions were presented, followed by the three experimental blocks of the memory recall task. In every one of the experimental blocks (*X3* marked in *red*), in each trial, participants studied a list of context-related and context-unrelated words, completed a working memory distraction task, and then were asked to indicate on a Likert scale how certain they were about their memory of a pair of words. Upon completing all trials (i.e., all three experimental blocks), the recording was stopped, and the participant was asked again if they were willing to send their recorded voices to the server (crossed out microphone symbol)

## Case study

To demonstrate the use of our framework, we conducted a relatively standard recognition-memory study. Participants were asked to remember lists of words presented one at a time during a study phase, and then were tested on their memory for those words, and their confidence in that memory, in a test phase. The study also included a manipulation of whether the study and test words came from a related category (body parts, cities, or countries), but this factor turned out to be mostly irrelevant for our purpose, and will be discussed in just one of the coming sections. Since the role of this experiment is as a case study in the use of our framework, we will only provide the experimental design features pertinent to this aim, and leave a more detailed description of the experiment to the Supplementary Materials.

**Fig. 3:**
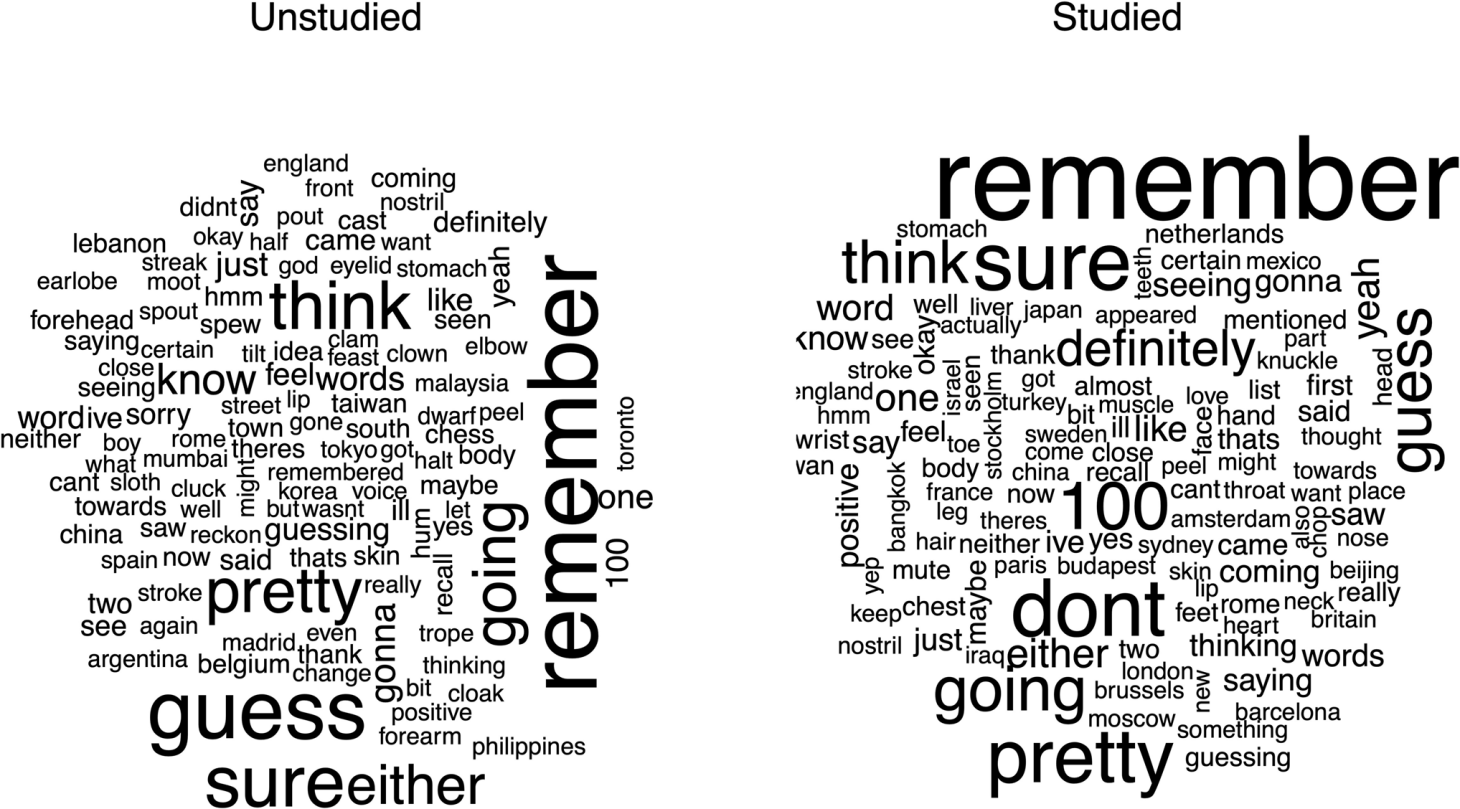
Word clouds for verbal reports studied and unstudied trials. Note: The size of the words in the cloud represents the frequency of the words appearing in the verbal reports; the larger and bolder it is displayed, the higher the words’ frequency

## Methods

Thirty-one participants were first asked to activate their microphones and grant permission for recording. After giving consent, they were then given general instructions explaining the task they were about to undertake. The instructions also included a statement that encouraged participants to use the think-aloud method. In short, they were asked to talk about how they made their decisions. They were asked to speak clearly and naturally, and were reassured not to worry about how they phrased their responses (for the exact wording, see the Instructions section in the Supplementary Materials). We will talk about the importance of such instructions in the Discussion section.

The main structure of the experiment was a study phase, distraction task, and test phase (see Fig. 2). In the study phase, participants were shown a set of 32 items that they were instructed to remember for a later test. During the distraction phase, to ensure that no studied items were still in working memory, participants performed a working-memory task for about 3 min. Finally, participants completed a test phase, where they were shown two items and were asked to indicate which of the two items was presented in the preceding study phase, and to indicate their confidence in that judgement using a slider that was marked to go from “guess” to “pretty sure” to “100% positive”. Crucially, some test trials contained two words that had not been studied earlier, while the vast majority of trials were such that one of the words was previously studied. This study-test phase cycle was repeated three times for each participant before the experiment was finished. Finally, after they completed the experiment, the recording stopped, and participants were asked whether they still consented to sending their recordings to our server.

## Results

### Descriptive statistics

#### Behavioral data

Participants’ performance in the memory task is not of particular interest here, and so it suffices to say that the experiment yielded pretty ’typical’ results. Namely, people were able to correctly indicate the items that were presented during the study phase with reasonable accuracy: they correctly identified “old” (previously studied) items on an average of .76 trials (SD = .075). Participants also gave a confidence rating on a scale of –1 to 1, where 0 is indifference between the two test items and –1 and 1 are complete certainty about either the incorrect or correct item. The confidence ratings were calibrated with the experimental task, in the sense that they were less confident (M = .06, SD = .12) when neither of the test items were presented in the study phase (i.e., both test items were unstudied), than when one of the two items was presented earlier (M = .52, SD = .13). Finally, participants’ confidence ratings were calibrated with their own memories, such that the magnitude of their confidence ratings for incorrect responses was lower (M = .43, SD = .19) than for correct responses (M = .78, SD = .11).

#### Verbal reports

Before we evaluate the various components of our framework, we want to give a quick insight into the types of things that participants talked about during the experiment. To do this, we present simple word clouds for all verbal report data from test phase trials. We made word clouds separately for test trials that included a word that was studied earlier (i.e., studied trials) and when both test words were not studied earlier (i.e., unstudied trials).

Figure 3, left cloud) shows that participants frequently used words indicating greater reliance on memory (e.g., “remember”, “know”) and higher confidence (e.g., “definitely”, “pretty sure”, and “sure”) on studied trials, but spoke as if they were uncertain (e.g., “guess”, “think”) on unstudied trials (see Fig.  3, right cloud). The word clouds also contain a lot of various nouns, and in almost all cases, these are the study items. This result makes sense, since looking at the raw verbal report data reveals that it was very common for participants to speak aloud the two test items presented on each trial.

We will now go through and describe how we used each of the components of our framework. Where possible, we will also provide a brief assessment of the effectiveness of the corresponding component.

### Recording

In our study, the framework generated a total of 533 .wav files per participant. Across all test trials, where participants were asked to choose between two words, the average length of the .wav files was 6.08 s (SD = 5.02) and the average file size was 1.18 MB. During the study trials, where participants were shown the words with a fixation-cross in between, the timing of the trials was fixed at 2.5 s and as expected the recordings duration were on average 2.45 s long (SD = .034) and the mean data size per trial was .46 MB (SD = .12 MB).

#### Effectiveness test

The effectiveness of the recording depends largely on the quality of participants’ microphones, the length of their utterances, and how much they enunciate. We, the authors, listened to a decent sample of the recordings, and we shared a vague impression that the recordings were mostly good enough to be usable, but there were certainly participants whose audio data could reasonably be discarded. However, we do note that we did not exclude any such participants from what follows, as we wanted the quantities we report to reflect the properties of the raw data.

**Table 4:** Effectiveness test for voice transcription

Label	Consistency rate	
	All trials	Test trials
“Perfect match”	.36	.19
“Good enough”	.69	.74
“Cutoff”	.19	.19
Word error rate (WER)	.3	.3

Note. *All trials* include trials from both the study phase and the test phase (refer to Methods for details). *Test trials* recordings consist of trials where participants rated their confidence in the familiarity of one of two words. Since this phase was not limited by time, compared to the study phase, the recordings were typically longer. *Word error rate (WER)* represents the metric used to evaluate the consistency between humans and model’s transcription

### Transcription

The second functionality of the software involved transcribing participants’ recordings (see Fig.  1, Output Box 4). We used the *large* version of the OpenAI Whisper model that has 1550 million parameters, was trained on 680,000 h of labeled speech data annotated using large-scale weak supervision (Radford et al., 2022a). We used the large version because the smaller versions produced noticeably, and often laughably, poor transcription performance (its inadequacy was evident from simply looking at the transcribed text in the .csv output files).

#### Effectiveness test

We compared the software’s transcriptions with those by human-raters to assess the accuracy and reliability of the software, providing an evaluation of the model’s transcription capabilities (Radford et al., 2022b). To do so, we selected a 5% sample from test trials and 5% from study trials across three blocks for each of the 31 participants, resulting in 404 test trials and 295 study trials. These sampled trials were then evaluated by the authors of the manuscript, who acted as the human raters for this test.

The evaluators listened to each of their sampled trials and created their own transcription into text. The rater then provided a subjective evaluation of whether the alignment between their transcription and that of the *Whisper* model was ’sufficiently aligned’ (good enough) (e.g., Model transcription: “Kiev, tricking Kiev”, human rater’s transcription: “Kiev, chicken Kiev”). The rater could also indicate whether the recording was ’cutoff’, meaning that the recording was not complete, and so it was only possible to transcribe the truncated part of what was spoken.

A summary of how often the match between the two transcriptions was perfect (i.e., exactly the same), ’good enough’, and ’cutoff’ is given in Table 4. It should become immediately clear why we included a subjective judgement of ’good enough’ in our analysis, as a perfect match was rather rare. Differences between human and LLM transcriptions were considerably more likely for test trials, where the utterances were typically much longer than for study trials, and so allowed for more possible errors. However, two things were clear from listening to the audio recordings and comparing the two transcriptions. First of all, the errors were typically unimportant from the perspective of what was meant by the speaker, which is most clearly seen by the fact that ’good enough’ ratings, if anything, are higher for test trials than study trials. Second, doing the transcriptions from the audio recordings was very obviously an error-prone process, even for human raters. Oftentimes, when the two transcriptions were inconsistent, it was also not particularly clear what was truly spoken, and hence whether either of the transcriptions was correct. However, most transcription errors were minor and did not distort the speaker’s intended meaning. This is best captured by the ’good enough’ rating – arguably the most relevant metric for downstream applications like utterance labeling. Transcriptions in this category, while not exact matches, preserved the core meaning and yielded nearly 70% alignment with human judgments. Thus, subsequent analyses remain valid.

**Fig. 4:**
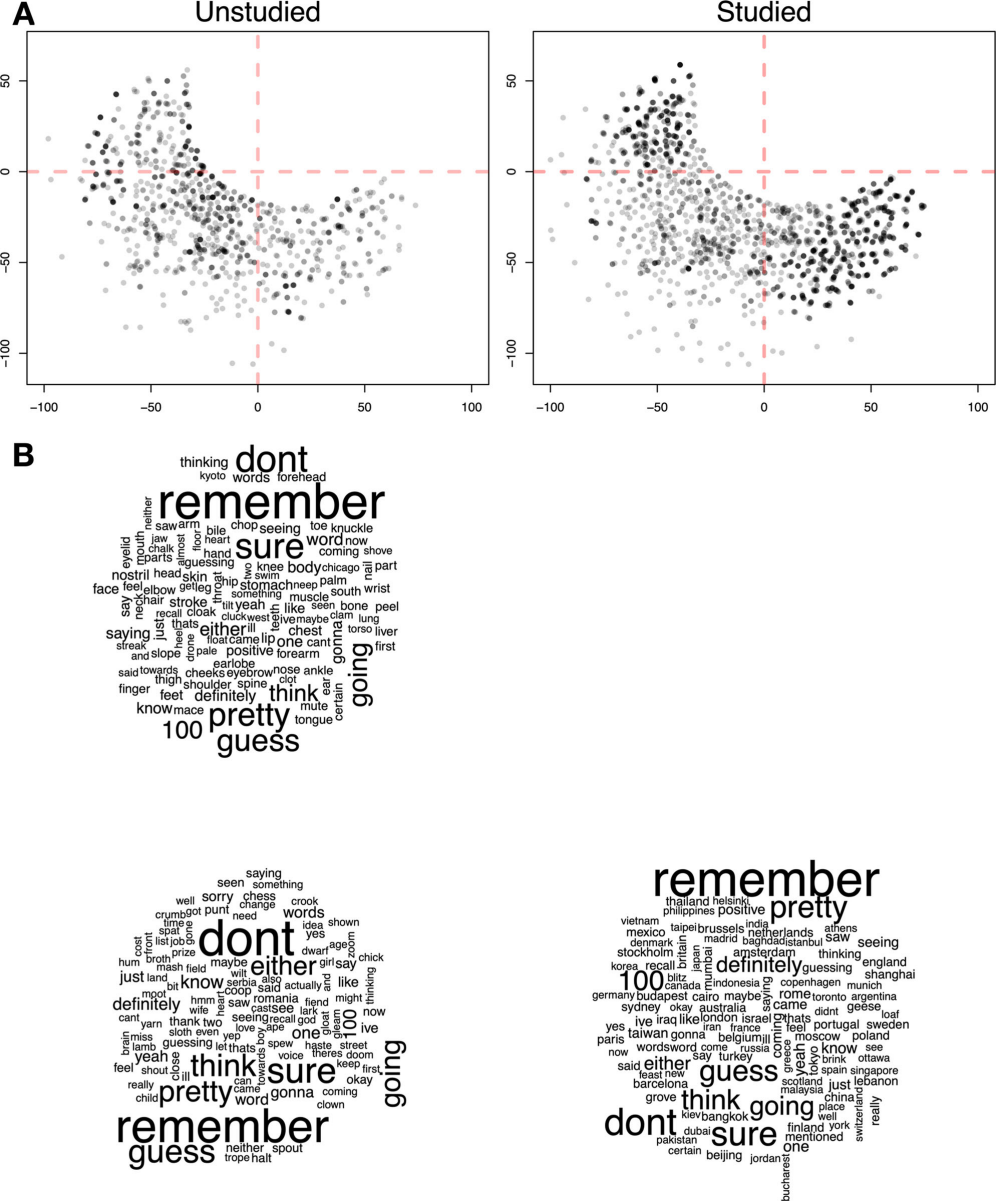
Dimensionality reduction applied to text data uttered by participants during experimental trials. Each *point* represents a unique utterance, mapped onto a two-dimensional space, with *dashed red lines* at the 0 mark to reveal patterns and clustering based on the content and context of the speech. The *dashed red lines* separate the data at the 0 mark to explore potential clusters. The left plot corresponds to the “unstudied” trial type, where participants encountered novel words, while the right plot shows the “studied” trial type, where participants were previously exposed to the words. The bottom subfigures represent word clouds, highlighting the most frequently uttered words present in each quarter of the plots where data was clustered

Acknowledging the subjective nature of our ’good enough’ measure, we also used the *word-error rate* as a more quantitative measure of the difference between the two transcriptions. This metric is widely used to evaluate the performance of speech recognition and machine translation systems. For instance, if the reference sequence is “I need to buy Apple” and the system transcribes it as “I need buy apples”, the error rate is calculated as .2 or 20%, reflecting one error (omission of “to”) in a sequence of five words. The results in Table 4 suggest that this transcription model yields roughly three errors for every ten words it transcribes.

twoDuring the human-based transcription process, we observed that Whisper frequently inserted filler phrases such as “thank you” in response to silent segments – an issue that is consistent with known limitations of the model and one that artificially inflated the word error rate (WER). To address this, we implemented a preprocessing step using the silero voice activity detection (VAD) model (Team, 2024), which reliably detects and excludes non-speech intervals prior to transcription. This adjustment led to a marked improvement in transcription quality, reducing the WER to 0.28 and increasing the “good enough” match rate to 81.6%. These enhancements are particularly valuable in experimental settings where occasional silent recordings can distort downstream analyses. To address this issue and streamline our processing pipeline, we have now integrated WhisperX into our end-to-end, jsPsych-based framework. WhisperX (Bain et al., 2023) improves upon the original Whisper (Radford et al., 2022a) model by incorporating a voice activity detection (VAD) module, achieving significantly faster transcription speeds (up to  70x), and reducing GPU memory usage – making it a more efficient and robust solution for handling audio data in behavioral experiments (see https://github.com/tehillamo/AutoV-LLM/blob/main/data_pipeline/speech2text.py)

To further improve transcription accuracy, we applied a post-processing step using ChatGPT, a large language model, to correct Whisper’s initial transcriptions. We used a tailored system prompt, which can be adjusted for various experimental settings (e.g., language, task type, or participant group). To enhance model performance, we provided contextual information – specifically, the list of study words used in the experiment – within the prompt (referred to as<Words used in the Study>). This step is critical, as participants often verbalize study words in isolation and without linguistic context (e.g., “Oh, I saw knee”), making accurate recognition challenging for ASR systems. Supplying the model with the actual list of words participants have seen constrains its vocabulary and helps disambiguate otherwise ambiguous utterances. Using this contextualized correction approach (see the full prompt we used in the Appendix), we observed a notable reduction in word error rate (WER). The overall WER dropped to .23. When excluding single-word transcriptions – which tend to lack interpretive value and are often noisier – the WER further improved to .18. Calculating WER per transcription (i.e., per row), we observed a median WER of .05, indicating that the average WER was inflated by a small number of highly inaccurate transcriptions. Closer inspection revealed that these outliers were often due to Whisper inserting extra words or confusing phonetically similar terms (e.g., "lamb" instead of "lamp")

Lastly, to improve transcription quality even further, future work may benefit from fine-tuning ASR models like Whisper on task-specific datasets. Such fine-tuning would allow the model to better adapt to the linguistic and contextual features of the experimental setup, improving recognition of short and semantically poor utterances. However, this approach requires a sufficient quantity of high-quality training data, which may not always be feasible to obtain.

### Embeddings

The third functionality of the software involves embedding sentences into 384-dimensional arrays (Fig.  1 Output Box 5) using the *Sentence Transformer* model ’all-MiniLM-L6-v2’ (Reimers & Gurevych, 2019).

#### Effectiveness test

The performance of LLMs is something that LLM developers are intensely monitoring, and the relative performance of all models is continuously changing. At the time of writing (September, 2024), the model we used was ranked 107th out of 477 on the ’Hugging Face’ leaderboard (for a complete list of models, see https://huggingface.co/spaces/mteb/leaderboard), which provides a comprehensive comparison of models, highlighting their versatility, effectiveness, and strengths or weaknesses in various contexts. The model we used was higher on the leaderboard when it was chosen for inclusion in the framework, and by the time this manuscript is read by others, we expect it will have slid even further down the rankings.

### Dimensionality reduction

In the fourth step, the software applies dimensionality reduction to the sentence embeddings, originally in a 384-dimensional format (see Box 6 in Fig. 1). While the framework offers two options, PCA (linear) and t-SNE, we show the output of just the latter. We reduced the embeddings using this method to two dimensions. The distribution of utterances on each trial, broken down according to whether there were only unstudied items or a studied item present, is plotted in Panel A of Fig. 4.

The goal of this analysis is to show that these reduced embeddings work in a way that is not so different from the well known and widely used method of principal component analysis (PCA), especially in behavioral science research involving self-report questionnaires. In those cases, researchers use PCA to find patterns in how people respond to different questions. Then, by looking at which questions group together, they can figure out what those groups represent. We take a similar approach here with the verbal reports. After reducing the dimensionality of the embeddings created from spoken utterances, we can look at the words in each area of the plot to understand what those dimensions might represent. This helps us uncover patterns in how participants talk, and reveals meaningful clusters based on the content of their speech.

#### Effectiveness test

Looking at the reduced embeddings in Fig. 4, it appears that there is no systematic difference depending on whether the words were studied or not. However, this does not mean that the reduced embeddings did not have any systematic structure. To gain some understanding of what these new dimensions represent, we used word clouds to summarize the different sections of the reduced space and plotted the results in Panel B of Fig. 4.

Looking at the word clouds, we see that words about confidence or certainty occur often in all three of the quadrants in which there are data (the fourth, upper-right quadrant consistent of only ’missing’ data, where the participant had said nothing during the recording). Furthermore, they seem to occur with roughly the same relative frequency, consistent with the fact that there are no systematic differences across unstudied and studied items.

The reduced embeddings seem to have captured differences in utterances not about certainty, but about the meaning of the nouns spoken. Namely, we see a lot of body parts in the top left quadrant, while the bottom right corner contains a lot of cities and countries. This result is unsurprising, given that half of the words in each study list were from one particular category: either body parts, countries, or cities. As such, this result reflects the fact that during the test phase, participants would typically refer to the words on screen while thinking aloud – e.g., “I definitely don’t remember seeing Iraq”. In other words, the dimensionality-reduction method has discovered the systematic structure that we built into the study and test items we used in our experiment.

### Text analysis

We decided that the most suitable text-analysis method for this experiment was the labeling pipeline, as the text was too brief for summarization or keyword extraction. We supplied the pipeline with the transcribed text from participants along with a set of candidate labels, listed in Table 5. We now analyze the output of this pipeline, the most likely label for the text from each trial.

**Table 5:** Mean confidence error - Effectiveness of labeling

Label	Rank
absolutely uncertain	0
very uncertain	1
somewhat uncertain	2
a little uncertain	3
a little certain	4
somewhat certain	5
very certain	6
absolutely certain	7

Note. Coding scheme for calculating mean confidence rate for labels

**Fig. 5:**
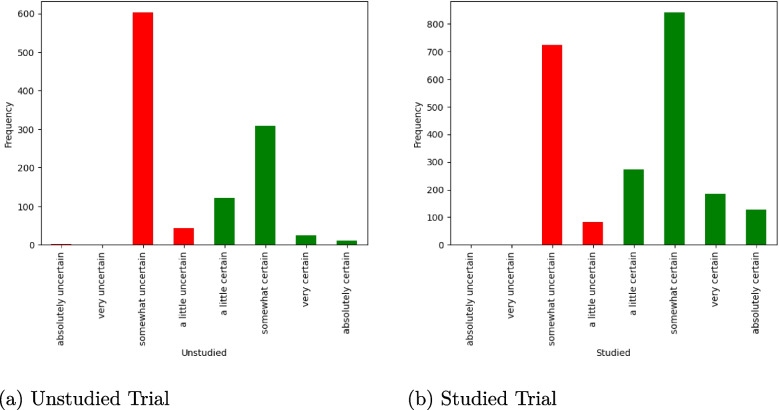
Frequency of label use. Note. The *bars* represent the frequency of certainty in participants’ verbal reports transcribed from two experimental trial types: ’unstudied’ and ’studied’. The *x*-axis represents the reports based on certainty levels ranging from ’absolutely uncertain’ to ’absolutely certain’. *Red bars* represent a meta label of ’uncertainty’, while *green bars* represent ’certainty’

In Fig. 5 we plot the frequency of label use, separated by whether the trial had a previously studied word present in the test trial (i.e., studied) or not (unstudied). As a visual aid, we color-coded the eight labels according to two rough categories: ’certain,’ and ’uncertain,’ using green and red colors, respectively. From the higher frequency of green bars in panel *b*, it appears that participants use more certain phrases on studied trials than unstudied trials.

#### Effectiveness test

To test the effectiveness of the labeling model’s output, we compared the model’s labels to those made by human raters, in a similar way we did for transcription evaluation earlier. A random sample of 10% of the “test” trials was given to human raters (again, these were the authors of the manuscript). The raters were presented with the transcribed text and were asked to choose the most appropriate label from the list of candidate labels that was given to the model.

A summary of the alignment between the model and human labels is presented in Table 6. We used three metrics to assess the functionality of labeling. Initially, we looked at how often the label assigned by the model and by human raters were identical. We call this measure the “overall accuracy”, and was 36% for our sample, where chance performance would be 12.5% (since there were eight labels). Like the transcription process, some of the distinctions among the labels were rather minor, and so we also looked at whether the labels were categorically similar. Following the color coding scheme in Fig. 5, both human and model labels were dichotomized into “certain” and “uncertain”, after which the agreement rate between the model’s labels and those chosen by humans was 82%. As a final way of comparing the labels, we assigned the eight labels the values 1 to 8, reflecting the increase from absolute uncertainty to absolute certainty (see Table 5). Using these numerical ranking values, we calculated the mean absolute error (MAE) between the model’s predicted labels and the human labels. The MAE was approximately 1, suggesting that the model’s predictions are typically off by one class (see Table 6).

**Table 6:** Effectiveness test for labeling

Test	Consistency rate
Overall accuracy	.36
Binary accuracy	.82
Mean confidence difference (MAE)	1.03

Note. All methods represent the metric used to evaluate the consistency between humans and model’s transcription. *Overall accuracy* represents a perfect match between the human and the models’ assigned labels. *Binary accuracy* refers to the match between the less nuanced labeling method, namely, the more rough classification of labels as either ’uncertain’ or ’certain’. *Mean confidence difference* refers to the average deviation of the model (here, it is by around one class)

## Discussion

In this paper, we introduce a newly developed framework that works within the jsPsych platform, designed to facilitate the collection and analysis of verbal reports in both offline and online studies. It automates numerous aspects of the collection of verbal report data, offers a range of possible analyses, and is readily expandable to incorporate new functionality. We used a case study as a proof-of-concept for the framework, and, where possible, provided relatively crude evaluations of the efficacy of various components of the framework. We will break the following discussion into two parts – first focusing on issues that came up while we developed the framework, and second, on the issues that arose when we used the framework during the case study.

**Table:** 

Trial no.	Transcribed text	RT	Embeddings	TSNE1	TSNE2	Highest similarity
100	West and geese I don’t	10532	[5.8e-02, -2.5e-02, -1.0e-02, 2.0e-02, ...]	30.77354	-56.467598	(’uncertain’, 0.29)
104	Pretty sure it’s spout.	10113	[-1.8e-02, -4.3e-02, -3.8e-02, 2.2e-02, ...]	-52.297226	-16.752245	(’certain’, 0.17)
520	I think Canada was there	3092	[9.6e-02, 9.6e-02, -3.2e-02, -9.2e-03, ...]	-2.408133	27.510927	(’uncertain’, 0.31)
516	China or geese	18038	[1.5e-02, 5.2e-02, -1.7e-02, 6.5e-02, ...]	5.694883	8.234329	(’unsure’, 0.29)
496	Float. Pretty sure. Float.	5669	[1.9e-02, 1.7e-02, -8.5e-02, 3.8e-02, ...]	-49.64332	-17.625269	(’pretty sure’, 0.24)
292	I don’t remember what I said.	14267	[-7.6e-02, 3.0e-03, 4.4e-02, 1.2e-01, ...]	5.032587	-36.959827	(’unsure’, 0.35)
484	Sweden I do have	2836	[6.7e-02, 4.3e-02, -2.5e-02, -4.0e-02, ...]	-37.824234	-55.96386	(’unsure’, 0.412)
448	I do remember seeing Taiwan.	5540	[9.6e-02, 4.1e-02, -1.2e-02, 4.5e-02, ...]	-30.39851	-80.51197	(’unsure’, 0.28)

### Developing the framework

#### Modularity and adaptability

The current framework was designed with modularity as its main objective. This means all components, including text-analysis pipelines, models, and their sizes, are adjustable. We viewed such flexibility as necessary because the models and methods available will rapidly become more sophisticated and potentially more affordable, which will enhance performance across the various functionalities (e.g., embeddings, transcription, and labeling). Furthermore, while our goal was to package together a core functionality that would be useful for most researchers – recording, transcription, and embeddings – we expect that the requirements of any individual research project will be highly variable.

Arriving at a version of the framework that would accompany this manuscript, and that we used in our case study, involved a number of choices. In our analyses, we chose specific models – such as the *Whisper model* (Radford et al., 2022a) for transcription, the *SentenceBERT model* (Reimers & Gurevych, 2019) for embedding, and the *Zero-shot model* for labeling – as well as various text analysis tools and techniques. These choices were based on practical considerations like whether the models were open source, whether the models could be run locally or needed a server, whether using the models cost money, where data would be stored, how large the data files produced would be and how much it would cost to store them, as well as other privacy issues (which we will return to shortly). Such questions, and more, will go into the choices about whether certain components in our framework need to be changed or replaced. The default set of methods and models we provide here was chosen to keep costs down, and hence it is likely that more accurate or better performing methods are available.

As of release, some of the components of our models have limitations. For instance, for the transcription task, our choice of the *Whisper model* (Radford et al., 2022a) in its current version has difficulty in accurately recognizing words from verbal reports that are brief, lack coherence, or contain grammatical irregularities. During the preparation of this manuscript, we became aware of a newly released ASR model that more effectively handles empty recordings and requires less computational overhead. In response, we updated our framework to support this model and documented the necessary adjustments in our GitHub repository. Users can now easily substitute the default ASR module with alternatives such as *WhisperX* or *NVIDIA’s Parakeet*. The repository includes worked examples demonstrating how to initialize, configure, and integrate these models within the pipeline (see https://github.com/tehillamo/AutoV-LLM/blob/main/readme.md). Detailed documentation and inline comments guide users through modifying the ASR component (https://github.com/tehillamo/AutoV-LLM/blob/main/data_pipeline/speech2text.py), ensuring the framework rem-ains extensible and adaptable to emerging tools.

These examples illustrate how the modular structure of our pipeline supports seamless integration of new models and ensures long-term adaptability. As transcription models continue to advance, the framework remains equipped to incorporate improvements in sensitivity and accuracy.

We also expect dimensionality reduction techniques to improve in the near future. One promising example is to use an autoencoder, which is a neural network that uses multiple hidden layers to learn to reproduce the high-dimensional data it is fed. The hidden layers of the autoencoders are of increasingly smaller dimensionality, and hence compress data into simpler representations. Since implementing this technique requires training a neural network model, we chose not to include it in our framework, but it is the kind of analysis that we might expect to become common in the near future.

#### Data privacy

One overarching concern during the development of the framework was the consideration of data privacy. To that end, we have implemented a number of particular mechanisms. Firstly, participants are required to confirm that they are being recorded. After the study is completed, participants must again confirm the use of their data. If a participant refuses, all recordings and data will be deleted from the server. Additionally, if a participant closes their browser window, their data will also be deleted as they were unable to confirm the use of their data. Each participant’s data is anonymized using a Universally Unique Identifier (UUID) that is created specifically for them. Finally, everything can be processed locally, so that we can avoid using any third-party services like Google or OpenAI, where it is not always clear whether the data is being kept or used.

#### Timing

One of the most difficult decisions researchers must make when collecting verbal-report data is when it should be elicited. For us, that meant determining how best to record and segment the verbal recordings. We chose to record for the entirety of the experiment because that seemed to allow for the vast majority of use cases we could imagine. For example, the researcher can instruct participants to speak whenever they want, or they could prompt participants to respond at particular times (e.g., after a behavioral response has been made, but before the next trial). In the latter case, a lot of what is recorded might be silence, but such irrelevant portions of the recording/transcriptions can be ignored during the data analysis phase.

The only complication in using continuous recording is that the user of the framework must consider and control how the recordings will be segmented in the output data files. For instance, if there is a reason to believe that participants might provide useful information while viewing a fixation cross, then the appropriate change to the jsPsych code must be made to make sure that any spoken text during the fixation cross is included as part of a jsPsych ’trial’. This way, both the stimulus presentation and the following fixation cross are treated as a single trial for data segmentation, recording, and transcription. Similarly, if the researcher wants to add some time for a verbal report after a behavioral response, they need to add some time to the experiment in jsPsych and perhaps have participants push a button to proceed (or advance after some predetermined time).

#### Evaluating the efficacy of the framework

One of the goals of our case study was to evaluate how well the framework would work as a user. We began this evaluation entirely informally, in the sense that we listened to samples of audio, read transcriptions, looked at the outputs of analyses with different settings, etc., until we had a sense that things were working well. For the manuscript, we came up with slightly more regimented ways to test things, in which we took samples of text and audio, and generated our own transcriptions and labels. The reader will have already presumably surmised that we did not intend these to be rigorous benchmarks or tests. Rather, we report on the efficacy of the framework for two reasons. First, we simply wanted to provide a proof-of-concept demonstration that the methods work. Second, we thought that such methods could give the reader an idea of how they might provide their own internal, informal checks that things are working as intended.

The reason for not carrying out careful and rigorous tests of this release version of the framework is that, because of the speed with which the specific models being used are developed and thus outdated, it would largely be a waste of time and effort. We expect that by the time this manuscript is published, users will be able to use newer and better transcription models. Furthermore, any performance we were able to record would be specific to the nature of our experiment (i.e., timing of recordings, the nature of the spoken utterances, etc.), and would be unlikely to generalize to other use cases for the framework.

Our relatively informal approach to testing the efficacy of our framework is only possible because of an interesting property of verbal-report data, which is that it is relatively easy to check whether things are working properly. That is, unlike the vast majority of dependent variables, all researchers have a well-developed understanding of the spoken word and how to know what it means. As such, anyone can simply look at the outputs of the framework, or what was done in a given analysis, and check whether it makes sense. The fact that verbal reports are more inherently ’transparent’ is a peculiar advantage, which makes it easier to criticize than many other dependent variables, such as fMRI or EEG signals. For example, other researchers can easily understand and critique the choice of labels we used, and use their own intuitions about whether a language model has made the same kind of label assignment as they would have. For other variables, it is much harder to validate analyses as sensible or valid. We hope that this property makes it easier than is typical to develop good methods of analysis, and draw sensible conclusions from resultant data.

### Lessons from the case study

#### Noisy data

One thing that is immediately obvious is that verbal reports are noisy. As its name implies, the think-aloud method can yield anything. As much as we would like them to, participants need not clearly articulate how they solved the task at hand. However, we are used to this as researchers, as it is no different from the fact that behavioral responses need not be made for the reasons that we expect or hope. In our case study, we also had noise in our verbal data because some reports were too short, were truncated by trial segmentation, poor microphones, or the utterances simply lacked any coherent explicable meaning.

As researchers, we can take steps to reduce the noise in verbal reports, and two kinds of strategies seem possible. First, we can improve the analysis/models. To some extent, this will happen naturally, since over time, we have already seen models become increasingly good at finding the signal in noisy data, even in shorter utterances. We can also adapt our analyses. For example, we had many trials during the study phase of the memory task in which participants just read the to-be-remembered word aloud. If accurate transcription was important for this aspect of our study, then we could have fine-tuned a transcription model to be sensitive to the study items.

Second, we could also increase the accuracy of transcription and embeddings by increasing data volume – whether through longer verbal reports. Longer verbal reports provide more context, enabling current models to better correct unclear words and “regularize” responses by replacing unusual words with more common ones. Clear instructions throughout the experiment can encourage participants to give more detailed responses. Ensuring that the structure of the experimental design permits sufficient time for participants to fully express their thoughts before moving on can also help to reduce the number of truncated responses. For example, we might have reduced the number of truncated responses by requiring participants to press a button to confirm that they were finished talking about the current trial. As mentioned earlier, the modularity of jsPsych and our framework supports these adaptations. Researchers can decide how to define jsPsych ’trials’ (i.e., what should be included in the trials) and can place instructions and buttons as needed.

Lastly, it’s also important to note the variability in the quality of our collected recordings. In some trials, it was difficult to discern precisely what was said due to the presence of background noise. This quality variation is expected, especially in online experiments. Using high-quality microphones and encouraging clear speech will improve the clarity of verbal report recording and transcriptions. For studies that require high transcription accuracy, conducting experiments offline in a lab setting with standardized equipment can ensure consistent audio quality.

#### Embeddings

When working with embeddings for verbal reports, researc-hers face a trade-off between using token-level (word-by-word) embeddings and sentence-level embeddings. Token-based models (e.g., Bert-base-uncased, Roberta-base) generate contextual embeddings for each individual token, capturing fine-grained linguistic features. This can be especially useful for identifying subtle nuances in language – for example, distinguishing between “I might have seen it” and “I’m sure I saw it,” where a small change in wording conveys a different level of certainty. However, because these models produce one embedding per token, an additional step is required to aggregate these into a single sentence representation (e.g., via mean pooling, using the [CLS] token, or applying attention-based pooling). This step introduces variability depending on the aggregation strategy and can influence downstream performance.

In contrast, sentence-embedding models (e.g., Sentence-BERT, MPNet, all-MiniLM) are specifically trained to produce a single fixed-length vector that captures the overall meaning of a sentence. These models are often fine-tuned using sentence-pair tasks (e.g., semantic similarity), making them more robust for applications where utterance-level comparisons, classification, or clustering are required. While this compression inevitably results in some loss of detail, sentence embeddings offer a practical and reproducible approach that is well-suited for many behavioral science applications – particularly when the goal is to analyze larger sets of verbal data efficiently.

In our framework, we use the full-dimensional sentence embeddings for classification tasks and apply dimensionality reduction only when needed for visualization or exploratory analysis. We prefer this approach because, while all embedding strategies involve some information loss, converting verbal reports into vector representations is essential for enabling systematic, quantitative analysis. To help users better understand the differences between these approaches, we recommend trying this tokenizer demo: https://tiktokenizer.vercel.app/ to see how text is broken down at the word level, and this embedding visualizer: https://huggingface.co/spaces/GIZ/embedding_visualisation to explore how sentence embeddings are structured in practice.

##### Fine tuning

In addition to the out-of-the-box approaches to embedding and text classification we included, we encourage readers to explore fine-tuning both the SentenceBERT and BART-NLI models, as each offers distinct advantages. Fine-tuning SentenceBERT can enhance the quality of sentence embeddings for tasks such as clustering or semantic similarity, while fine-tuning BART-NLI improves performance in classification tasks, especially when using zero-shot approaches. Adapting these models to your specific dataset makes them more accurate, context-aware, and better suited to the nuances of your domain.

The full script for fine-tuning SentenceBERT is available at https://github.com/tehillamo/AutoV-LLM/tree/main/data_pipeline/example_scripts. The script begins by reading a CSV file and filtering the data to ensure quality. It then applies a technique called Contrastive Tension, which trains the model to pull semantically similar sentences closer together and push dissimilar ones farther apart in the embedding space. The *MiniLM* model serves as the starting point, and after fine-tuning is completed, the output is tested to generate sentence embeddings that are better aligned with the input data.

#### Dimension reduction

Our analyses focused on how people talked about their confidence depending on whether they were trying to remember words they had studied and those they had not. We expected that the dimensionality reduction analysis would reveal systematic differences in expressions of certainty, since we did observe people talking a lot about how confident they were. However, our analysis indicated that other aspects of the verbal reports were more important. Indeed, we found no systematic differences between studied and unstudied words, but rather found that the reduced dimensions captured variation in the meanings of the nouns spoken, rather than certainty about word recognition or the concept of context. This is not to say that the analysis was flawed, but it is a reminder that dimensionality reduction methods are theoretically agnostic and will find whatever structure is present. As such, the onus is on the researcher to design experiments that encourage people to speak more or less about certain things.

There are several effective strategies for managing the spoken content in verbal reports to increase the chances that the reduced embeddings will capture what a researcher intends. As previously discussed, researchers can direct participants to focus on specific topics of interest instead of allowing them to choose their topics freely. For instance, participants could be encouraged to talk about their confidence in their choices or explain how the associations between nouns (i.e., context) helped them with memory recall. We might also have asked participants not to say aloud the words they had to remember, however, this might have affected the way that people performed in the experiment, and so care must be taken with such instructions. Another approach is to alter the analyses, for example, by excluding from the dimensionality reduction the trials on which participants said the study words, or by replacing all such words with a single word. We also could have looked at dimensionality reduction solutions with more dimensions, as it might have been that the third dimension was about confidence.

#### Transcription & text analysis pipelines

Our case study results showed a relatively poor alignment between human raters and machine/model transcriptions in terms of the precise labels that were chosen. Indeed, we saw similar discrepancies when we used an even greater variety of possible labels (in analyses not presented here). However, in many cases, the choice between specific candidate labels, as a human rater, felt largely arbitrary, and so we would expect similar discrepancies in human classification. More importantly, it was rare that the machine classifier would make a completely incongruous classification (i.e., where it appears to have lost all semantic meaning). For the sake of analysis that uses aggregation, perfect transcription is hardly crucial. Indeed, we accept some level of disagreement among human raters. One benefit here is that we can overcome such noise with substantially larger data sets, and so labeling can still preserve the semantic meaning, despite the inevitable discrepancies with human judgement.

While we can largely ignore problems of noisy labeling, we should be sensitive to biases in machine-based analyses. For example, in our labeling analyses, we had the algorithm classify all utterances according to their degree of certainty. Now, a classifier will make such a determination, regardless of whether or not the participants actually talked about their confidence on a given trial. As such, it is possible for these analyses to produce misleading results, since certain words or phrases might be systematically assigned certain labels, regardless of whether it makes any sense. For example, the classifier in our analysis might have consistently labelled country or city names as ’high certainty’. This would have produced a misleading bias in the analysis, which might have caused us to conclude that people were more confident when making decisions about proper nouns, say. There is no prescriptive method for ruling out such biases; they can only minimized by researchers who remain vigilant when checking that their conclusions are not due to trivial features of an automated analysis.

To help mitigate this, our code includes the option to set a mi-nimum matching score, allowing users to filter out low-confi-dence classifications (see script "text_classification" on https://github.com/tehillamo/AutoV-LLM/blob/main/data_pipeline/text_classification.py). Additionally, if concerns remain or if the assigned labels consistently receive low confidence scores, we suggest a practical diagnostic approach: introducing a random, unrelated category and observing whether it captures a substantial portion of the text. This simple method can help identify potential issues with label refinement or signal that the current label set may require further adjustment.

We chose to use a "zero-shot" text classification technique in our case study. However, there are currently three alternative approaches that can be considered. The first is Natural Language Inference (NLI), which involves determining whether a hypothesis logically follows from a given premise by classifying the relationship as entailment, contradiction, or neutral. For example: *Premise*: “The cat is sleeping on the couch.” *Hypothesis*: “The animal is resting indoors.” *classification* Entailment (the premise supports the hypothesis). A key strength of this approach is that models are explicitly trained for inference tasks, which typically results in strong performance. Additionally, NLI models are relatively lightweight in terms of resource requirements. However, a considerable weakness is that performance can be highly dependent on how the classification labels are phrased or framed.

A second approach relies on embeddings. In this embed-ding-based method for zero-shot text classification, vector representations are computed for both the input text and the category labels. Classification is then performed by measuring the semantic similarity between these embeddings – typically using metrics like cosine similarity. This approach is highly efficient and well-suited for large-scale applications where speed is a priority. However, it tends to struggle with more abstract or nuanced category labels. Additionally, embedding-based models are not explicitly trained for classification tasks, which can limit their accuracy in some contexts.

A third approach to consider when deciding on a zero-shot classification is the *prompt-based (generative)* method. This technique uses large language models (e.g., GPT-4, T5) to perform zero-shot text classification by framing the task as a natural language prompt. The model then generates a classification label based on its understanding, without requiring explicit training on the specific task. This approach is highly flexible and can incorporate few-shot examples to improve accuracy. It is particularly well suited for tasks that require reasoning or adaptation to new contexts. However, its primary drawback is the significant computational cost.

#### Model selection for multilingual use

For researchers working with other languages, we recommend exploring alternative models. One popular alternative is the *xlm-roberta-large-xnli* model (for details see https://huggingface.co/joeddav/xlm-roberta-large-xnli), which sup-ports 15 popular languages. That said, where possible, we advise using monolingual models, as they tend to be better optimized for performance in a specific language.

#### Generalizability and applicability

Not every experimental task is well suited for verbal reports. Some tasks are naturally more compatible with this method than others. This has been discussed extensively in the seminal work by Ericsson and Simon (1980), and more recently by Ostrovsky et al. (2024). For example, intuitively, tasks performed in rapid succession or those that are highly restricted in trial completion time may result in little to no verbal output during task performance. Other tasks, such as our memory task described in the case study, allow more time for verbalization. However, even in these settings, if participants are not explicitly instructed to speak continuously, they may still produce brief or fragmented responses – as we observed in our study. Nevertheless, even shorter responses can be valuable – especially when researchers invest time and effort into fine-tuning their models, which can lead to meaningful and reliable analyses. Naturally, tasks that encourage longer and more detailed verbal responses are the best candidates, as both transcription and text analysis benefit from richer contextual information. One final point worth considering is whether asking for verbal reports will have a considerable effect on the way that the participant performs the task. Such concerns can be somewhat alleviated by comparing the data collected with verbal reports to existing behavioral data or appropriate control conditions that do not collect verbal reports.

## Conclusion

We have presented a practical framework tailored for gathering and evaluating verbal reports in computer-based psychology experiments. It provides researchers with the ability to deal with many of the biggest issues with verbal-report data. Most importantly, work that has typically required human judgment can now be done by machine-learning-based methods, thus making the transcription and analysis of verbal reports much more scalable. We showed that the framework works using a simple memory experiment. The code we provide can be used as is, meaning that psychology researchers should be able to start incorporating verbal reports into their experiments. However, the framework is modular, meaning it can be updated and expanded as methods are improved and discovered, and the full potential of quantifying verbal data is realized.

The data that support the findings of this study are available at https://osf.io/xa7rk/. Data are available upon submission.

## Supplementary Information

Below is the link to the electronic supplementary material.

Supplementary file 1 (tex 13 KB)

## Data Availability

The dataset generated and analyzed for the current study are available in the "AutoV-LLM" repository under https://osf.io/xa7rk/.

## Appendix

Here is the full prompt we used to improve our transcription analysis:

## Footnotes

1 https://github.com/tehillamo/AutoV-LLM/blob/main/beginners_guide.pdf

2 this benchmark was published after the implementation of our framework.

## Code availability

The analysis scripts as well as the software’s scripts are publicly available at https://github.com/tehillamo/AutoV-LLM.
